# Intraoperative oliguria does not predict postoperative acute kidney injury in major abdominal surgery: a cohort analysis

**DOI:** 10.1590/2175-8239-JBN-2019-0244

**Published:** 2020-08-10

**Authors:** Rita Inácio, Joana Gameiro, Solange Amaro, Mafalda Duarte

**Affiliations:** 1 Hospital Prof. Doutor Fernando da Fonseca, Divisão de Anestesiologia, Lisboa, Portugal.; 2 Centro Hospitalar Lisboa Norte, Departmento de Medicina, Divisão de Nefrologia e Transpalntação Renal, Lisboa, Portugal.; 3 Hospital Prof. Doutor Fernando da Fonseca, Lisboa, Portugal.

**Keywords:** Acute Kidney Injury, Oliguria, General Surgery, Treatment Outcome, Predictors, Lesão Renal Aguda, Oligúria, Cirurgia Geral, Resultado do Tratamento, Preditores

## Abstract

**Background:**

Acute kidney injury (AKI) is a common complication in patients undergoing major abdominal surgery and is associated with considerable morbidity and mortality. Several studies investigating the association between intraoperative urine output and postoperative AKI have shown conflicting results. Here, we investigated the association of intraoperative oliguria with postoperative AKI in a cohort of patients submitted to elective major abdominal surgery.

**Methods:**

This was a single-center retrospective analysis of adult patients who underwent elective major abdominal surgery from January 2016 to December 2018. AKI was defined according to the serum creatinine criteria of the KDIGO classification. Intraoperative oliguria was defined as urine output of less than 0.5 mL/kg/h. Risk factors were evaluated using multivariate logistic regression analysis.

**Results:**

A total of 165 patients were analyzed. In the first 48 h after surgery the incidence of AKI was 19.4%. Postoperative AKI was associated with hospital mortality (p=0.011). Twenty percent of patients developed intraoperative oliguria. There was no association between preexisting comorbidities and development of intraoperative oliguria. There was no correlation between the type of anesthesia used and occurrence of intraoperative oliguria, but longer anesthesia time was associated with intraoperative oliguria (p=0.007). Higher baseline SCr (p=0.001), need of vasoactive drugs (p=0.007), and NSAIDs use (p=0.022) were associated with development of intraoperative oliguria. Intraoperative oliguria was not associated with development of postoperative AKI (p=0.772), prolonged hospital stays (p=0.176) or in-hospital mortality (p=0.820).

**Conclusion:**

In this cohort of patients we demonstrated that intraoperative oliguria does not predict postoperative AKI in major abdominal surgery.

## Resumo

**Introdução:**

Lesão renal aguda (LRA) é uma complicação comum em pacientes submetidos a grandes cirurgias abdominais, e está associada a considerável morbimortalidade. Vários estudos investigando a associação entre débito urinário intraoperatório e LRA pós-operatória mostraram resultados conflitantes. Neste trabalho investigamos a associação de oligúria intraoperatória com LRA pós-operatória em uma coorte de pacientes submetidos à grandes cirurgias abdominais.

**Métodos:**

Análise retrospectiva de centro único envolvendo pacientes adultos submetidos à grandes cirurgias abdominais, de janeiro de 2016 a dezembro de 2018. A LRA foi definida segundo critérios de creatinina da KDIGO. Definimos oligúria intraoperatória como débito urinário inferior a 0,5 mL/kg/h. Fatores de risco foram avaliados por análise de regressão logística multivariada.

**Resultados:**

Analisamos 165 pacientes. Nas primeiras 48 horas após a cirurgia, a incidência de LRA foi de 19,4%. LRA pós-operatória foi associada à mortalidade hospitalar (p = 0,011). 20% dos pacientes desenvolveram oligúria intraoperatória, sem associação com comorbidades preexistentes. Não houve correlação entre o tipo de anestesia e oligúria intraoperatória; entretanto, maior tempo de anestesia esteve associado à oligúria intraoperatória (p = 0,007). Maior creatinina sérica (Cr) inicial (p = 0,001), necessidade de drogas vasoativas (p = 0,007) e uso de AINEs (p = 0,022) foram associados à oligúria intraoperatória. Oligúria intraoperatória não foi associada ao desenvolvimento de LRA no pós-operatório (p = 0,772), à permanência hospitalar prolongada (p = 0,176) ou à mortalidade intra-hospitalar (p = 0,820).

**Conclusão:**

Demonstramos que a oligúria intraoperatória não prediz LRA pós-operatória em cirurgias abdominais de grande porte.

## Background

Acute kidney injury (AKI) is characterized by a rapid decrease in renal function ^(^ 1 ^)^. In hospitalized patients, up to 40% of AKI cases are in the surgical context ^(^ 1 ^)^. Major abdominal surgery is the second most common surgical setting associated with AKI ^(^ 2 ^,^ 3 ^)^. AKI after major abdominal surgery is multifactorial and results from an intricate interaction of hemodynamic, toxic, and inflammatory factors ^(^ 3 ^,^ 4 ^)^.

AKI has a significant impact in short- and long-term outcomes. Indeed, it has been associated with progression to chronic kidney disease (CKD), increased cardiovascular risk, longer hospital stays, and increased mortality risk ^(^ 5 ^-^ 8 ^)^. Given the impact of post-operative AKI, it is critical to detect predictors and early markers of AKI in this setting so as to timely prevent and manage this complication ^(^ 9 ^)^.

Various factors have been implicated in the risk of postoperative AKI ^(^ 10 ^,^ 11 ^)^. Urine output is a sensitive and early marker of acute kidney injury and is associated with poor outcomes ^(^ 12 ^,^ 13 ^)^. However, the role of intraoperative oliguria as a predictor of AKI reveals conflicting results in the literature. In fact, it is common belief that intraoperative oliguria might not reflect kidney hypoperfusion as it is also affected by several hormones, namely aldosterone, atrial natriuretic peptide, and antidiuretic hormone, which are released during surgery and anesthesia ^(^ 3 ^)^.

In this study, we investigated the association of intraoperative oliguria with postoperative AKI defined according to the serum creatinine (SCr) criteria of the KDIGO classification, in a cohort of patients submitted to major abdominal surgery.

## Materials and Methods

### Study Design

This was a retrospective analysis of clinical data of patients who underwent elective major abdominal surgery at the Department of Surgery of Hospital Prof. Doutor Fernando da Fonseca from January 2016 to December 2018. The study was approved by the Ethical Committee at Hospital Fernando da Fonseca, in agreement with institutional guidelines. Informed consent was waived by the Ethical Committee due to the retrospective and non-interventional nature of the study.

### Participants

All patients aged 18 or older who underwent elective major abdominal surgery of the Department of Surgery of Hospital Fernando da Fonseca from January 2016 to December 2018 were eligible for this study.

Major abdominal surgery was defined as intraperitoneal approach performed under general anesthesia, with a predictable length of hospital stay of at least two days. ^(^ 14 ^,^ 15 ^)^ For patients with more than one surgery, only the first procedure was considered. In patients with multiple hospital admissions, only the first one was considered. After surgery the patients were transferred to the post-anesthesia care unit.

Exclusion criteria were CKD patients already on renal replacement therapy, patients who underwent renal replacement therapy the week before surgery, patients who had less than two determinations of SCr during hospital stay, patients who were discharged from hospital less than two days after the procedure, patients who underwent emergency surgery, and patients without intraoperative diuresis monitoring.

### Variables

All variables were collected from electronic patient clinical records, including intraoperative data recorded by the anesthesiologist. All scores and formulas were calculated based on raw clinical data.

The analyzed variables included demographic characteristics (age, gender, weight, height), preoperative clinical characteristics, type of anesthesia (balanced anesthesia, total intravenous anesthesia, combined epidural, and general anesthesia), physical status according to the American Society of Anesthesiologists (ASA) score ^(^ 16 ^)^, surgical APGAR score ^(^ 17 ^)^, preoperative serum hemoglobin, preoperative neutrophils, lymphocytes and platelets, baseline SCr, preoperative serum glucose, duration of anesthesia, use of fluids (colloids - hydroxyethyl starch, gelatin and albumin 5%; crystalloids - sodium chloride 0.9%, Ringer’s lactate and polyelectrolyte solution), intraoperative urine output, intraoperative blood transfusions and use of vasoactive drugs, intraoperative use of furosemide and intraoperative use of non-steroidal anti-inflammatory drugs within the last hour to manage postoperative pain, postoperative intensive care unit (ICU) stay, length of stay, postoperative AKI and mortality.

Regarding preoperative clinical characteristics, the comorbidities registered were diabetes mellitus (diagnosed according to the American Diabetes Association criteria ^(^ 18 ^)^), hypertension (diagnosed according to the seventh report of the Joint National Committee ^(^ 19 ^)^), cardiovascular disease (including chronic heart failure, cardiac ischemic disease, and history of transient ischemic attack or stroke), chronic obstructive pulmonary disease (COPD) including emphysema and chronic bronchitis, and malignancy. For cardiovascular disease and COPD, indication on clinical records of previous diagnosis was considered sufficient.

Blood transfusions were done in patients with active bleeding or hemodynamically unstable or when the serum hemoglobin level was below the 7 to 8 g/dL range ^(^ 20 ^)^ or, in older patients and in patients with coronary artery disease, bellow 10g/dL.

### Definitions

Only AKI developing in the first 48 h after surgery was considered to be attributed to the surgical procedure. AKI was diagnosed using the Kidney Disease Improving Global Outcome (KDIGO) classification based on SCr criteria, as an increase in SCr of 0.3 mg/dL within 48 hour-periods, or an increase in SCr of 1.5 times baseline, which is known, or within the prior 7 days, or a decrease in urine output to less than 0.5 mL/kg/h for 6 hours ^(^ 21 ^)^. (Table 1) Pre-operative SCr was considered baseline SCr.

**Table 1:** Patient’s characteristics at baseline and according to the development of AKI.

Characteristic	Baseline (n=165)	No AKI (n=133)	AKI (n=32)	p value
Age (years)	69.2±14.0	67.4±13.8	76.7±12.4	0.001
Gender (male) – n (%)	96 (58.2)	77 (57.9)	19 (59.4)	0.879
Weight (kg)	67.0±14.3	67.4±14.9	65.5±11.6	0.502
Height (cm)	165.0±7.0	165±7.1	165±6.7	0.849
Ideal body weight (IBM) (kg)	58.0±8.0	57.9±8.1	58.3±7.7	0.848
*Co-morbidities – n (%)*				
Hypertension	100 (60.6)	76 (57.1)	24 (75)	0.063
Diabetes	38 (23)	31 (23.3)	7 (21.9)	0.863
CVD	62 (37.6)	45 (33.8)	17 (53.1)	0.043
COPD	10 (6.1)	5 (3.8)	5 (15.6)	0.012
CKD	16 (9.7)	9 (6.8)	7 (21.9)	0.010
Malignancy	121 (73.3)	94 (70.7)	27 (84.4)	0.116
ASA ≥ 3	102 (61.8)	75 (56.4)	24 (75)	0.087
*Baseline Laboratory*				
Baseline SCr (mg/dL)	0.95±0.55	0.88±0.4	1.24±0.9	0.001
Baseline hemoglobin (g/dL)	11.1±1.8	11.2±1.9	10.7±1.6	0.171
Preoperative glycemia (mg/dL)	119.4±55.9	118.6±57.9	122.6±47.4	0.719
*Anesthesia type – n (%)*				
Balanced	111 (67.3)	86 (64.7)	25 (78.1)	0.327
Total intravenous anesthesia	1 (0.6)	1 (0.8)	0 (0)	0.327
Combined	53 (32.1)	46 (34.6)	7 (21.9)	0.327
*Intra-operative factors*				
Anesthesia time (h)	4.2±1.7	4.2±1.8	4.4±1.5	0.491
Use of vasoactive drugs – n (%)	36 (22)	25 (18.8)	11 (34.4)	0.058
Use of ephedrine – n (%)	67 (40.6)	50 (37.6)	17 (53.1)	0.108
Use of furosemide – n (%)	31 (18.8)	25 (18.8)	6 (18.8)	0.995
Use of NSAIDs – n (%)	51 (30.9)	38 (28.6)	13 (40.6)	0.185
Blood transfusions – n (%)	37 (22.4)	28 (21.1)	9 (28.1)	0.389
Use of crystalloid only – n (%)	146 (88.5)	118 (88.7)	28 (78.1)	0.846
Crystalloid infusion volume (L)	3.1±1.3	3.07±1.4	3.26±1.2	0.471
Use of both crystalloids and colloids – n (%)	19 (11.5)	15 (11.3)	4 (12.5)	0.846
Colloid infusion volume (L)	0.8±0.2	0.68±0.35	0.66±0.41	0.700
Fluid volume (L)	3.2±1.5	3.2±1.5	3.4±1.3	0.385
Surgical Apgar	7.4±1.7	7.4±1.9	7.4±1.2	0.893
Intra-operative urinary output – mL/k/h	1.85±1.79	1.9±1.9	1.6±1.3	0.427
Intra-operative urinary output adjusted to IBW– mL/k/h	2.1±2.1	2.2±2.2	1.7±1.4	0.299
Intra-operative oliguria – n (%)	34 (20.6)	28 (21.1)	6 (17.8)	0.772
Intra-operative oliguria (IBW) – n (%)	24 (14.5)	19 (14.3)	5 (15.6)	0.847
Intra-operative oliguria (lower threshold) – n (%)	32 (19.4)	8 (6.0)	3 (9.4)	0.494
*Outcomes*				
AKI – n (%)	32 (19.4)			
Renal replacement therapy – n (%)			4 (12.5%)	
LOS in hospital (days)	33.8±26.7	31.6±26.1	43.2±27.7	0.027
Hospital mortality – n (%)	27 (16.4)	17 (12.8)	10 (31.3)	0.011

AKI- acute kidney injury, CVD- cardiovascular disease, COPD- chronic obstructive pulmonary disease, CKD- chronic kidney disease, ASA- American Society of Anesthesiologists score, SCr- Serum creatinine, NSAIDs- Nonsteroidal anti-inflammatory drugs, LOS- length of stay

Ideal body weight (IBW) was calculated according to the Devine formula ^(^ 22 ^)^.

Intraoperative oliguria was defined as urine output of less than 0.5 mL/kg/h ^(^ 23 ^)^.

### Outcomes

Development of AKI, in-hospital mortality, and length of hospital stay were assessed.

### Statistical Methods

Continuous variables are presented as the mean ± standard deviation and categorical variables as the total number and percentage of cases for each category. After grouping participants according to the development of postoperative AKI, the variables of both groups were compared using Student’s t-test for normally distributed continuous variables and chi-square test for categorical variables.

Firstly, all variables underwent univariate analysis to determine statistically significant factors that may have contributed to AKI and mortality. Only variables that significantly differed between AKI and non-AKI groups were used in the multivariate analysis using the Cox logistic regression method. An adjusted multivariate analysis to pre-operative, intra-operative, and post-operative factors was conducted. Data are expressed as odds ratios (OR) with 95% confidence intervals (95%CI). A sensitive analysis was carried out to adjust the definition of intraoperative oliguria to the calculated ideal body weight. Statistical significance was defined at a p-value (p) <0.05. Analyses were performed with the statistical software package SPSS 21.0 for Windows.

## Results

### Participants

**Figure 1:**
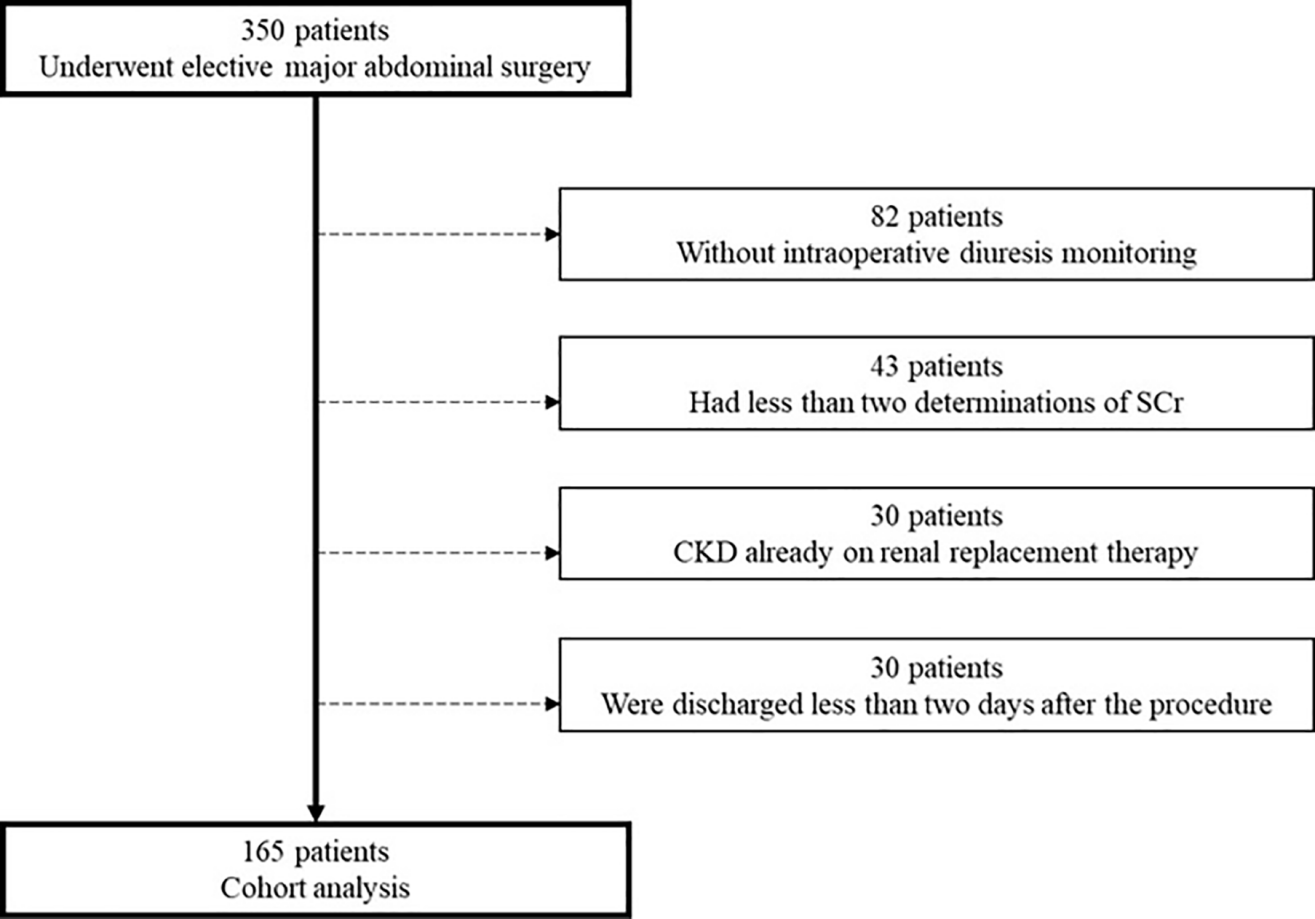
Flow-chart of patient selection. SCr- Serum creatinine, NSAIDs- Nonsteroidal anti-inflammatory drugs, OR- odds ratio

We identified 350 eligible patients. Of these, 185 were excluded as follows: 82 patients due to lack of monitoring of intraoperative diuresis, 43 had less than two determinations of SCr, 30 had been hospitalized for less than 48 hours and 30 patients were chronic kidney disease patients on dialysis (Figure 1).

We focused on a cohort of 165 patients. Demographic, preoperative, intraoperative, and postoperative patient variables and outcomes are described in Table 2.

**Table 2:** Univariate and multivariate analysis of factors predictive of AKI.

	AKI			
	Unadjusted OR (95% CI)	P-value	Adjusted OR (95% CI)	P-value
Age	1.06 (1.03-1.10)	0.001	1.06 (1.02-1.10)	0.006
CKD	3.86 (1.30-11.31)	0.014	2.36 (0.73-7.65)	0.153
CVD	2.2 (1.01-4.84)	0.046	0.98 (0.39-2.47)	0.970
COPD	4.74 (1.28-17.50)	0.020	4.26 (1.02-17.45)	0.047

AKI- acute kidney injury, CVD- cardiovascular disease, CKD- chronic kidney disease, OR- Odds ratio

In this cohort of patients who underwent elective major abdominal surgery, mean age was 69.2±14.0 years, the majority were males (58.2%), mean height was 165.0±7.0cm, mean weight was 67.0±14.3, and mean IBW was 58.0±8.0kg. Sixty percent of patients were hypertensive, 23% were diabetic, 37.6% had cardiovascular disease, 6.1% had COPD, and 9.7% had CKD. Seventy-three percent of patients had malignancy. Baseline SCr was 0.95±0.55mg/dL and baseline Hb was 11.1±1.8g/dL. The mean ASA score was 2.7±0.8. In 67.3% of patients balanced anesthesia was performed. Mean anesthesia time was 4.2±1.7 hours. During surgery, 22% of patients required vasoactive drugs, ephedrine was administered in 40.6% of patients, furosemide in 18.8% of patients, and NSAIDs in 30.9%. Twenty two percent of patients required blood transfusions (mean volume administered was 511.9±471.8 mL). Crystalloids were administered in all patients, and in 11.5% of patients both colloids and crystalloids were administered. Mean surgical APGAR was 7.4±1.7 (Table 1).

In the first 48 h after surgery, 32 patients (19.4%) developed AKI. The majority of patients were classified as KDIGO stage 1 (43.8%), 31.2% KDIGO stage 3 and 25% as KDIGO stage 2. Four patients required renal replacement therapy (12.5%).

Patients with postoperative AKI were older (p=0.001, unadjusted OR 1.06 (1.03-1.10) p=0.001), and were more likely to have preexisting CVD (p=0.043, unadjusted OR 2.2 (1.01-4.84), p=0.014), COPD (p=0.012, unadjusted OR 4.74 (1.28-17.50), p=0.020) and CKD (p=0.010, unadjusted OR 3.86 (1.30-11.31), p=0.014). There was no difference between the type of anesthesia performed and AKI incidence. Concerning intraoperative factors, there was a trend for more vasoactive drug need (p=0.058). There was no association between the measured intraoperative urine output and AKI (Table 1).

In a multivariate analysis, only older age (p=0.001, unadjusted OR 1.06 (1.03-1.10) p=0.001, adjusted OR 1.06 (1.02-1.10), p=0.006), and COPD (p=0.012, unadjusted OR 4.74 (1.28-17.50), p=0.020, adjusted OR 4.26 (1.02-17.45), p=0.047) were associated with postoperative AKI (Table 2).

The patients had a mean length of stay in hospital of 33.8±26.7 days. The in-hospital mortality in this cohort of postoperative AKI patients was 16.4%. Mortality was higher in older patients (p<0.001), patients with pre-existent hypertension (p=0.001), CVD (p=0.003), COPD (p=0.003) or CKD (p=0.002), patients with higher baseline SCr (p<0.001) and lower baseline hemoglobin (p=0.006), patients who required intraoperative blood transfusions (p<0.001), patients with AKI (p=0.011), and patients who required renal replacement therapy (p<0.001). Additionally, mortality was higher according to the severity of AKI (KDIGO stage 1 - 18.8%, KDIGO stage 2 - 37.5%, KDIGO stage 3 - 40%, p=0.049 (Table 3).

**Table 3:** Demographic and clinical characteristics according to in-hospital mortality.

Characteristics	Survival (138)	Mortality (27)	p value
Age (year)	67.3±14.1	78.8±9.0	<0.001
Gender (male) – n (%)	78 (56.5)	18 (66.7)	0.328
Weight (kg)	66.8±14.2	68.5±15.2	0.561
Height (cm)	165.1±7.0	164.7±6.9	0.830
Ideal body weight (IBM) (kg)	57.9±8.1	68.5±15.2	0.919
*Co-morbidities – n (%)*			
Hypertension	76 (55.1)	24 (88.9)	0.001
Diabetes	29 (21.0)	9 (33.3)	0.164
CVD	45 (32.6)	17 (62.9)	0.003
COPD	5 (3.6)	5 (18.5)	0.003
CKD	9 (6.5)	7 (25.9)	0.002
Malignancy	98 (71.0)	23 (85.2)	0.128
ASA ≥ 3	82 (59.4)	20 (74.1)	0.152
*Baseline Laboratory*			
Baseline SCr (mg/dL)	0.87±0.42	1.36±0.89	<0.001
Baseline Hemoglobin (g/dL)	11.3±1.7	10.2±1.9	0.006
Preoperative Glycemia (mg/dL)	120±58	116±43.6	0.752
*Anesthesia type – n (%)*			
Balanced	91 (65.9)	20 (74.1)	0.669
Total intravenous anesthesia	1 (0.7)	0 (0)	0.669
Combined	46 (33.3)	7 (25.9)	0.669
*Intra-operative factors*			
Anesthesia time (h)	4.2±1.8	4.1±1.6	0.747
Use of vasoactive drugs – n (%)	30 (21.7)	6 (22.2)	0.970
Use of ephedrine – n (%)	60 (43.5)	7 (25.9)	0.089
Use of furosemide – n (%)	26 (18.8)	5 (18.5)	0.969
Use of NSAIDs – n (%)	40 (28.9)	11 (40.7)	0.227
Blood transfusions – n (%)	24 (17.4)	13 (48.1)	<0.001
Use of crystalloid only – n (%)	125 (90.6)	21 (77.8)	0.057
Crystalloid infusion volume (L)	3.1±1.4	3.3±1.2	0.353
Use of both crystalloids and colloids – n (%)	13 (9.4)	6 (22.2)	0.057
Colloid infusion volume (L)	772.7±248.9	600.0±200	0.230
Fluid volume (mL)	3.2±1.5	3.6±1.4	0.170
Surgical APGAR	7.4±1.8	7.4±1.3	0.892
Intra-operative urinary output – mL/k/h	1.8±1.8	1.9±1.7	0.890
Intra-operative urinary output adjusted to IBW– mL/k/h	2.1±2.0	2.3±2.4	0.650
Intra-operative oliguria – n (%)	28 (20.3)	6 (22.2)	0.820
Intra-operative oliguria (IBW) – n (%)	19 (13.8)	5 (18.5)	0.522
*Outcomes*			
AKI – n (%)	22 (15.9)	10 (37.0)	0.011
Renal replacement therapy – n (%)	0 (0)	4 (14.8)	<0.001
LOS in hospital (days)	31.7±25.8	44.7±29.3	0.020

AKI- acute kidney injury, CVD- cardiovascular disease, COPD- chronic obstructive pulmonary disease, CKD- chronic kidney disease, ASA- American Society of Anesthesiologists score, SCr- Serum creatinine, NSAIDs- Nonsteroidal anti-inflammatory drugs, LOS- length of stay

### Intraoperative Oliguria And Postoperative AKI

Twenty percent of patients had intraoperative oliguria (mean urine output 0.3±1.1 mL/k/h). When adjusted to the IBW, 14.5% of patients had intraoperative oliguria (mean urine output 0.4±0.2 mL/k/h).

There was no association between preexisting comorbidities and development of intraoperative oliguria. Mean baseline SCr was higher in patients who developed intraoperative oliguria (p=0.001, unadjusted OR 2.35 (1.21-4.54), p=0.011, adjusted OR 2.03 (1.01-4.12), p=0.048). There was no correlation between the type of anesthesia used and occurrence of intraoperative oliguria, but longer anesthesia time was associated with intraoperative oliguria (p=0.007, unadjusted OR 1.32 (1.07-1.61), p=0.008, adjusted OR 1.27 (1.00-1.62), p=0.053). Concerning intraoperative factors, intraoperative oliguria was more frequent in patients who required vasoactive drugs (p=0.007, unadjusted OR 3.43 (1.51-7.82), p=0.003, adjusted OR 2.71 (1.04-7.07), p=0.041) and in whom NSAIDs were administered (p=0.022, unadjusted OR 2.44 (1.12-5.30), p=0.025, adjusted OR 3.06 (1.28-7.35), p=0.012) (Table 4).

**Table 4:** Demographic and clinical characteristics according to intraoperative oliguria

Characteristics	No-oliguria (131)	Intraoperative oliguria (34)	p value
Age (year)	69.8±13.7	66.8±15.1	0.271
Gender (Male) – n (%)	72 (54.9)	24 (70.6)	0.100
Weight (kg)	64.9±12.9	75.4±16.5	0.000
Height (cm)	164.6±7.1	166.6±6.5	0.139
Ideal body weight (IBM) (kg)	57.5±8.1	60.0±7.2	0.105
*Co-morbidities – n (%)*			
Hypertension	76 (58)	24 (70.6)	0.181
Diabetes	27 (20.6)	11 (32.4)	0.147
CVD	45 (34.4)	17 (50)	0.093
COPD	8 (6.1)	2 (5.9)	0.961
CKD	10 (7.6)	6 (17.6)	0.079
Malignancy	96 (73.3)	25 (73.5)	0.977
ASA ≥ 3	77 (58.8)	25 (73.5)	0.115
*Baseline Laboratory*			
Baseline SCr (mg/dL)	0.89±0.5	1.2±0.6	0.003
Baseline Hemoglobin (g/dL)	11.1±1.8	11.2±2.0	0.693
Preoperative Glycemia (mg/dL)	117.7±52.0	125.9±69.3	0.457
*Anesthesia type – n (%)*			
Balanced	88 (67.2)	23 (67.6)	0.878
Total intravenous anesthesia	1 (0.8)	0 (0)	0.878
Combined	42 (32.1)	11 (32.4)	0.878
*Intra-operative factors*			
Anesthesia time (h)	4.0±1.7	4.9±1.7	0.007
Use of vasoactive drugs – n (%)	22 (16.8)	14 (41.2)	0.002
Use of ephedrine – n (%)	49 (37.4)	18 (52.9)	0.100
Use of furosemide – n (%)	21 (16.0)	10 (29.4)	0.075
Use of NSAIDs – n (%)	35 (26.7)	16 (47.1)	0.022
Blood transfusions – n (%)	31 (23.7)	6 (17.6)	0.454
Use of crystalloid only – n (%)	117 (89.3)	29 (85.3)	0.513
Crystalloid infusion volume (L)	3.1±1.4	3.0±1.3	0.780
Use of both crystalloids and colloids – n (%)	14 (10.7)	5 (14.7)	0.513
Colloid infusion volume (L)	0.76±0.25	0.67±0.24	0.775
Fluid volume (mL)	3.2±1.5	3.2±1.4	0.911
Surgical APGAR	7.5±1.6	7.1±2.2	0.223
Intra-operative urinary output – ml/k/h	2.2±1.8	0.3±1.1	0.000
Intra-operative urinary output adjusted to IBW– ml/k/h	2.5±2.1	0.4±0.2	0.000
*Outcomes*			
AKI – n (%)	26 (19.8)	6 (17.6)	0.772
Renal replacement therapy – n (%)	4 (3.1)	0 (0)	0.302
LOS in hospital (days)	32.4±23.4	39..4±36.8	0.176
Hospital mortality – n (%)	21 (16.0)	6 (17.6)	0.820

AKI- acute kidney injury, CVD- cardiovascular disease, COPD- chronic obstructive pulmonary disease, CKD- chronic kidney disease, ASA- American Society of Anesthesiologists score, SCr- Serum creatinine, NSAIDs- Nonsteroidal anti-inflammatory drugs, LOS- length of stay

In the multivariate analysis, higher baseline SCr, need of vasoactive drugs, and NSAIDs use remained associated with development of intraoperative oliguria, and there was a trend for the association of longer anesthesia time and intraoperative oliguria (Table 5).

**Table 5:** Univariate and multivariate analysis of factors predictive of intraoperative oliguria.

	Intraoperative oliguria			
	Unadjusted OR (95% CI)	P-value	Adjusted OR (95% CI)	P-value
Baseline SCr	2.35 (1.21-4.54)	0.011	2.03 (1.01-4.12)	0.048
Anesthesia time	1.32 (1.07-1.61)	0.008	1.27 (1.00-1.62)	0.053
Use of vasoactive drugs	3.43 (1.51-7.82)	0.003	2.71 (1.04-7.07)	0.041
Use of NSAIDs	2.44 (1.12-5.30)	0.025	3.06 (1.28-7.35)	0.012

The development of intraoperative oliguria was not associated with development of postoperative AKI (p=0.772), renal replacement therapy requirement (p=0.306), prolonged hospital stays (p=0.176), or in-hospital mortality (p=0.820) (Table 4).

A sensitive analysis was performed using a lower threshold for the definition of intraoperative oliguria as urine output lower than 0.3 mL/kg/h. In this analysis there was also no correlation between intraoperative oliguria and AKI (p=0.494).

## Discussion

In this retrospective cohort, we demonstrated that intraoperative oliguria did not predict the occurrence of postoperative AKI in patients submitted to elective major abdominal surgery. In past years, many studies have focused on the association between the development of intraoperative oliguria and postoperative AKI, nevertheless this association remains poorly defined.

Surgical patients are hemodynamically unstable, hypovolemic, have low cardiac output and elevated hormone and catecholamine levels, all of which, associated to the effects of general anesthesia, can influence diuresis. ^(^ 3 ^)^

Fluid depletion is multifactorial and is a result of nil-by mouth regimens and the loss of fluid due to the concomitant pathology, surgical bleeding and intravascular fluid losses, insensible losses, and extravasation of fluid out of the vascular compartment ^(^ 3 ^,^ 10 ^)^. The effect of general anesthesia impairs myocardial function and is associated with vasodilation, which further contributes to kidney hypoperfusion ^(^ 3 ^,^ 10 ^)^. Consequently, the renal response is afferent arteriole dilation and efferent arteriole vasoconstriction in order to maintain glomerular filtration, and activation of the renin-angiotensin system causes sodium and water retention and potassium loss ^(^ 3 ^,^ 10 ^,^ 12 ^,^ 13 ^)^. The increase in catabolic hormones and cytokines associated with surgery leads to antidiuretic hormone secretion and water retention. Thus, the apparent physiological response to surgery by reducing urine output does not support that oliguria could be a reliable intraoperative marker of postoperative AKI ^(^ 24 ^)^. Indeed, previous studies report the lack of association between intraoperative urine output and postoperative AKI ^(^ 25 ^)^.

In a study by Alpert et al. of 137 patients undergoing aortic reconstruction no significant correlation was found between intraoperative mean urine output or lowest hourly urine output and AKI ^(^ 26 ^)^. The same results were presented in a study by Knos et al. of patients submitted to aortic reconstruction ^(^ 27 ^)^. In a prospective analysis of 15,102 patients submitted to major non-cardiac surgery intraoperative oliguria defined as urine output less than 0.5m L/kg/h was not a predictor of AKI ^(^ 28 ^)^. These are consistent with the results presented in our study.

In contrast, more recent studies have demonstrated that intraoperative oliguria can predict postoperative AKI. Hori et al. conducted a prospective study of 579 cardiac surgery patients and reported that there was an independent correlation between low urine output of less than 1.5 mL/kg/h and postoperative AKI ^(^ 29 ^)^. These results have also been reproduced after major non cardiac surgery. In a retrospective cohort study of 3560 major abdominal surgery patients, Mizota el al. found that intraoperative oliguria defined as urine output of less than 0.3 mL/kg/h was a predictor of postoperative AKI. However, milder levels of oliguria, defined as urine output of 0.3 to less than 0.5 mL/kg/h were not associated with AKI ^(^ 30 ^)^. In our study we conducted a sensitivity analysis to define oliguria with a lower threshold of 0.3 mL/kg/h and still there was no correlation between intraoperative oliguria and AKI.

Interestingly, the duration of intraoperative oliguria might predict AKI development. In a retrospective analysis of 5894 patients submitted to major non-cardiac surgery intraoperative oliguria, defined as urine output of less than 0.5 mL/kg/h for more than 120 minutes, was associated with postoperative AKI. Furthermore, longer duration of intraoperative oliguria was significantly associated with the severity of postoperative AKI ^(^ 31 ^)^. However, the previous studies included emergency surgery patients, which has been reported in several studies as a predictive factor of AKI, and although its influence is still not consensual, could influence the results ^(^ 10 ^)^. Our study focused on elective surgery patients.

Fluid balance during surgery has also been associated with AKI development ^(^ 32 ^)^. The Restrictive Versus Liberal Fluid Therapy in Major Abdominal Surgery (RELIEF) trial, a multicenter randomized trial, enrolled 2983 patients undergoing major abdominal surgery. In this study, Myles et al. found that restrictive fluid therapy was associated with higher incidence of oliguria and postoperative AKI ^(^ 32 ^)^. However, a recent meta-analysis concludes that there is insufficient evidence to suggest that restrictive fluid management is associated with an increase in oliguria and AKI risk ^(^ 33 ^)^. In our cohort, fluid balance during surgery was goal-directed and there was no association between fluid balance and development of intraoperative oliguria or postoperative AKI.

A post hoc analysis study of the RELIEF trial reported that oliguria, defined as urine output of less than 0.5 mL/kg/h, had a low predictive ability for postoperative AKI. Indeed, it was the absence of oliguria that demonstrated a good predictive ability of not developing postoperative AKI ^(^ 34 ^)^.

To enhance the predictive ability of oliguria, a study by Kim and colleagues suggests that intraoperative oliguria in combination with a decrease in SvO_2_ can predict postoperative AKI more reliably than each of these factors alone, in a retrospective cohort of patients undergoing living donor liver transplantation ^(^ 34 ^)^. Considering the limitations of intraoperative oliguria and the conflicting results reported in literature, perhaps the combination of multiple intraoperative variables can increase the prediction of postoperative AKI.

Our findings are in conflict with most recent studies investigating intraoperative oliguria and AKI in non-cardiac surgery. In our study of patients submitted to major abdominal surgery, we demonstrated that the development of intraoperative oliguria was not associated with age, patient comorbidities, anesthesia type, fluid administration volume, or diuretic administration. Intraoperative oliguria developed more frequently in patients with higher baseline SCr, patients who required intraoperative vasoactive drugs, and NSAIDs use. This vulnerable group of patients were more prone to developing an intraoperative decrease of urine output. Nevertheless, we found that intraoperative oliguria was not correlated with AKI, length of hospital stay, or in-hospital mortality. Indeed, these findings question the role of intraoperative oliguria as a predictor of AKI.

However, there are some limitations that must be taken into account. Firstly, the single-center nature of our study limits the generalizability of the results. Second, the retrospective design with a moderately small cohort of patients may have overlooked some potential confounders with prognostic importance, such as intra-abdominal pressure, serum calcium, and serum uric acid. Indeed, only older age and COPD remained independent predictors of AKI in the multivariate analysis, which could signify that certain AKI risk factors were not identified in the small cohort of patients. Nevertheless, the fact that no correlation with AKI was identified in the sensitivity analysis restricting the criteria for intraoperative oliguria suggests that intraoperative oliguria may not be a consistent predictor of AKI.

Despite these limitations, our study has several notable strengths. To our knowledge, this is the first study focusing on elective major abdominal surgery reporting that intraoperative oliguria is not predictive of AKI development. We conducted a retrospective analysis focusing on major abdominal surgery patients and despite the retrospective nature of the study, most of the studied variables were registered as part of routine clinical practice on a daily basis and made accessible for analysis. Our results restore the previous evidence that intraoperative oliguria is not a reliable marker of postoperative AKI.

In conclusion, we demonstrated that intraoperative oliguria did not predict postoperative AKI in elective major abdominal surgery patients. The role of intraoperative oliguria as a predictive marker of AKI remains to be determined and further studies are required to conclude that intraoperative oliguria reversal protocols could avoid postoperative AKI.
